# Unveiling cathode catalysis of fluorinated electrolyte additives for high-performance Na-Cl_2_ batteries

**DOI:** 10.1093/nsr/nwaf333

**Published:** 2025-08-12

**Authors:** Qiuchen Xu, Shanshan Tang, Shuo Wang, Anrong Chen, Yan Wang, Shitao Geng, Bin Yuan, Chengxiao Zhang, Qianyun Chen, Zhaofeng Ouyang, Feng Zhu, Xiaoju Zhao, Hao Sun

**Affiliations:** Frontiers Science Center for Transformative Molecules, School of Chemistry and Chemical Engineering, Zhangjiang Institute for Advanced Study, Shanghai Jiao Tong University, Shanghai 200240, China; Frontiers Science Center for Transformative Molecules, School of Chemistry and Chemical Engineering, Zhangjiang Institute for Advanced Study, Shanghai Jiao Tong University, Shanghai 200240, China; Frontiers Science Center for Transformative Molecules, School of Chemistry and Chemical Engineering, Zhangjiang Institute for Advanced Study, Shanghai Jiao Tong University, Shanghai 200240, China; Frontiers Science Center for Transformative Molecules, School of Chemistry and Chemical Engineering, Zhangjiang Institute for Advanced Study, Shanghai Jiao Tong University, Shanghai 200240, China; Frontiers Science Center for Transformative Molecules, School of Chemistry and Chemical Engineering, Zhangjiang Institute for Advanced Study, Shanghai Jiao Tong University, Shanghai 200240, China; Frontiers Science Center for Transformative Molecules, School of Chemistry and Chemical Engineering, Zhangjiang Institute for Advanced Study, Shanghai Jiao Tong University, Shanghai 200240, China; Frontiers Science Center for Transformative Molecules, School of Chemistry and Chemical Engineering, Zhangjiang Institute for Advanced Study, Shanghai Jiao Tong University, Shanghai 200240, China; Frontiers Science Center for Transformative Molecules, School of Chemistry and Chemical Engineering, Zhangjiang Institute for Advanced Study, Shanghai Jiao Tong University, Shanghai 200240, China; Frontiers Science Center for Transformative Molecules, School of Chemistry and Chemical Engineering, Zhangjiang Institute for Advanced Study, Shanghai Jiao Tong University, Shanghai 200240, China; Frontiers Science Center for Transformative Molecules, School of Chemistry and Chemical Engineering, Zhangjiang Institute for Advanced Study, Shanghai Jiao Tong University, Shanghai 200240, China; Frontiers Science Center for Transformative Molecules, School of Chemistry and Chemical Engineering, Zhangjiang Institute for Advanced Study, Shanghai Jiao Tong University, Shanghai 200240, China; Frontiers Science Center for Transformative Molecules, School of Chemistry and Chemical Engineering, Zhangjiang Institute for Advanced Study, Shanghai Jiao Tong University, Shanghai 200240, China; Frontiers Science Center for Transformative Molecules, School of Chemistry and Chemical Engineering, Zhangjiang Institute for Advanced Study, Shanghai Jiao Tong University, Shanghai 200240, China

**Keywords:** chlorine battery, fluorinated additive, interphase layer, cathode catalysis

## Abstract

Rechargeable sodium-chlorine (Na-Cl_2_) batteries offer a promising solution for next-generation energy storage, due to their high electrochemical performance and reliance on abundant, cost-effective materials. The electrolyte typically comprises a mixture of aluminum chloride (AlCl_3_) and thionyl chloride (SOCl_2_), with the addition of sodium bis(fluorosulfonyl)imide (NaFSI) and sodium trifluoromethanesulfonimide (NaTFSI) as F-containing additives. These additives have been considered to form a fluorinated solid-electrolyte interphase layer on the Na metal anode to enhance cycling stability, a mechanism analogous to those observed in conventional alkali metal batteries. Here we reveal a previously unrecognized spontaneous reaction between these additives and AlCl_3_ in the electrolyte, producing AlF_3_ on the cathode. This enables facilitated NaCl/Cl_2_ oxidation due to the strong Lewis acidity of AlF_3_, and suppresses parasitic reactions. These findings not only correct the mechanism misunderstanding of fluorinated additives in rechargeable Na-Cl_2_ batteries but, in a broader context, open a new avenue for turning conventional anode protective additives into efficient cathode catalysts for high-rate and long-life energy storage solutions.

## Teaser

Fluorine-containing additives in sodiumchlorine batteries can form an efficient cathode catalyst to improve rate and cycling performance, opening a new avenue for high-rate, long-life rechargeable batteries.

## INTRODUCTION

**Figure 1.**
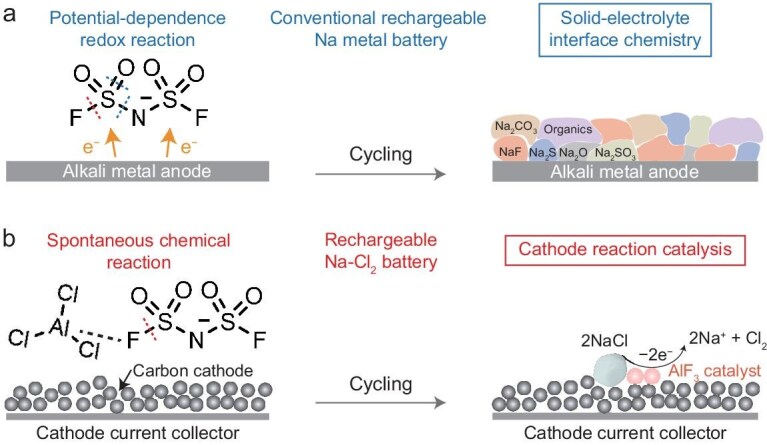
Schematic illustration of the working mechanisms of FSI^−^ anion in a conventional Na metal battery and Na-Cl_2_ battery. (a) Conventional rechargeable Na metal battery with a potential-dependence redox reaction for solid-electrolyte interphase (SEI) formation on Na metal anode through FSI^−^ anion decomposition. (b) Rechargeable Na-Cl_2_ battery with a spontaneous chemical reaction between the FSI^−^ anion and AlCl_3_ in the chloroaluminate electrolyte to form AlF_3_ at the carbon cathode, which further facilitates the oxidation of NaCl as an efficient Lewis-acidic catalyst.

The increasing demand for renewable energy has driven the pursuit of sustainable, high-performance energy storage solutions [1–7]. Among various candidates, rechargeable Na-Cl_2_ batteries have emerged as promising candidates due to the natural abundance and low cost of Na and Cl [8,9]. It can deliver a high specific capacity of 1200 mAh g^−1^ (based on the mass of carbon) at a high discharge voltage of ∼3.5 V, along with high-rate capability through a rational design of cathode materials as described in our recent work [10]. The core component of this system is the chloroaluminate electrolyte composed of AlCl_3_ and SOCl_2_, coupling with F-containing additives including NaFSI and NaTFSI. These additives are widely believed to decompose electrochemically at the anode/electrolyte interface, forming an F-rich solid-electrolyte interphase (SEI) layer, similar to mechanisms proposed in conventional Li and Na metal batteries (Fig. 1a) [11–15]. However, this assumption warrants re-evaluation due to the strong Lewis acidity of AlCl_3_, which may drastically react with fluorinated additives, leading to a fundamentally different mechanism from that observed in conventional alkali metal batteries. Clarifying the working mechanism of F-containing additives in rechargeable Na-Cl_2_ batteries not only addresses a fundamental challenge in this battery system, but also opens new avenues for designing advanced electrolyte and electrode materials with practical applications.

Here we show the reaction and evolution of fluorinated electrolyte additives in rechargeable Na-Cl_2_ batteries, involving a spontaneous chemical reaction with AlCl_3_ in the chloroaluminate electrolyte (Fig. 1b). Mechanistic studies indicate the cleavage of the S−F bond in FSI^−^ anions activated by AlCl_3_, with the formation of AlF_3_ following a reaction similar with the sulfur (VI) fluoride exchange (SuFEx) click chemistry [16,17]. The formed AlF_3_ shows negligible impact on Na plating/stripping reversibility at the anode. Instead, it can effectively facilitate the oxidation of NaCl as an efficient Lewis-acidic catalyst, thereby enhancing cathode reaction kinetics and reversibility. These findings not only enrich the understanding of F-containing electrolyte additives in alkali metal batteries, but also open a new avenue for rational design of electrode and electrolyte catalysts for high-performing Na-Cl_2_ batteries.

## RESULTS AND DISCUSSION

**Figure 2.**
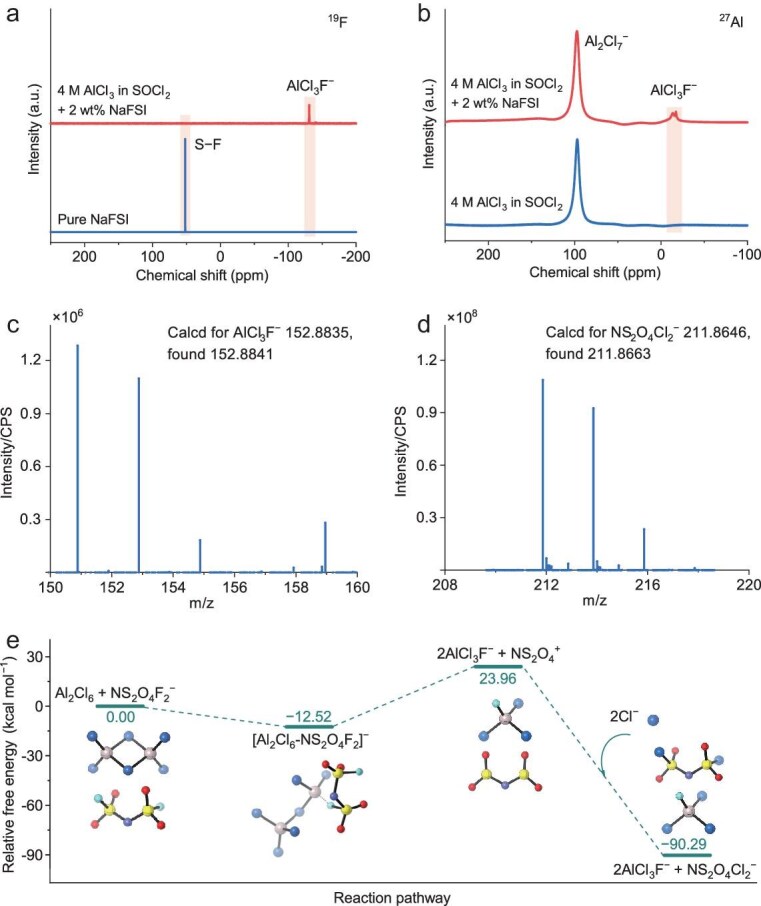
Characterizations of spontaneous reaction between NaFSI and AlCl_3_ in the chloroaluminate electrolyte. (a) ^19^F NMR spectra of pure NaFSI and the electrolyte comprised of 4 M AlCl_3_ in SOCl_2_ with 2 wt% NaFSI. (b) ^27^Al NMR spectra of the electrolytes comprised of 4 M AlCl_3_ in SOCl_2_ with and without 2 wt% NaFSI. (c, d) HRMS spectra of AlCl_3_F^−^ and NS_2_O_4_Cl_2_^−^ detected from the electrolyte comprised of 4 M AlCl_3_ in SOCl_2_ with 2 wt% NaFSI, respectively. (e) Gibbs free energy calculations of the electrolyte reaction between Al_2_Cl_6_ and NS_2_O_4_F_2_^–^ (FSI^–^). Al_2_Cl_6_ can coordinate with the FSI^−^ anion and cleave the S–F bond with the formation of NS_2_O_4_^+^ and AlCl_3_F^–^. The NS_2_O_4_^+^ cation further combines with Cl^−^ to form NS_2_O_4_Cl_2_^−^.

The spontaneous reaction between the FSI^−^ anion and AlCl_3_ was verified by nuclear magnetic resonance (NMR) and high-resolution mass spectrometry (HRMS) (Fig. 2a–d and Fig. S1). ^19^F NMR spectra of the electrolyte comprising 4 M AlCl_3_ in SOCl_2_ with 2 wt% NaFSI revealed complete disappearance of the signature peak of the S−F bond in NaFSI at 52 ppm (Fig. 2a) [18], indicating the complete cleavage of the S−F bond in NaFSI. Meanwhile, a new peak at −130 ppm appeared in the ^19^F NMR spectra, which aligned with the new peak at ∼−15 ppm in ^27^Al NMR spectra (Fig. 2b). The product was identified as AlCl_3_F^−^ according to HRMS (Fig. 2c), where the mass spectrum matched the characteristic isotopic signature. In addition, the formation of NS_2_O_4_Cl_2_^−^ was verified (Fig. 2d), originating from the spontaneous chemical reaction between the FSI^−^ anion and AlCl_3_. Density functional theory (DFT) calculations further indicate the spontaneity of the above reaction (Fig. 2e).

**Figure 3.**
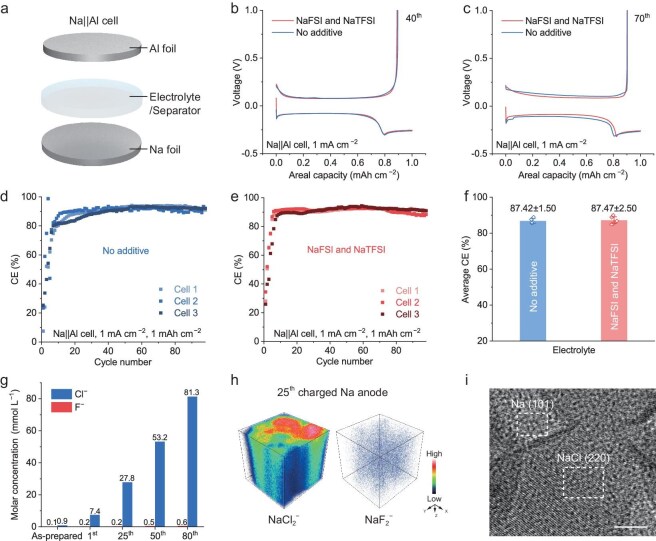
Electrochemical performance and interfacial chemistry of Na anode mediated by F-containing electrolyte additives. (a) Schematic illustration of a Na||Al cell. (b, c) Comparison of galvanostatic charge-discharge curves at the 40th and 70th cycles of the Na||Al cells using the AlCl_3_−SOCl_2_ electrolyte with and without F-containing additives, respectively. (d, e) Cycling performance of Na||Al cells using the AlCl_3_−SOCl_2_ electrolyte without (d) and with F-containing additives (e), respectively. The areal capacity and current density in (b–e) are 1 mAh cm^−2^ and 1 mA cm^−2^, respectively. (f) Statistical analysis of the average CE of the Na||Al cells using electrolytes with and without F-containing additives. (g) Molar concentrations of Cl^−^ and F^−^ on the anode at various cycle numbers detected by ion chromatography. (h) Three-dimensional distributions of NaCl_2_^−^ and NaF_2_^−^ secondary ion fragments constructed from a TOF-SIMS depth scan using a fully charged anode after 25 cycles. The charge capacity and current density are 1000 mAh g^−1^ and 1 A g^−1^, respectively. (i) High-resolution Cryo-TEM images of Na-plated Au grid. A Na/Au grid coin cell was assembled to allow Na deposition with an areal capacity of 0.02 mAh cm^−2^ at a current density of 0.2 mA cm^−2^. Scale bar, 5 nm.

The aforementioned reaction inspires us to validate the true mechanism of F-containing electrolyte additives in rechargeable Na-Cl_2_ batteries. We thus studied the impact of F-containing electrolyte additives on Na plating/stripping reversibility using Na||Al cells (Fig. 3a). The 4 M AlCl_3_ in SOCl_2_ electrolyte was used as a basic electrolyte (denoted as ‘no additive’ electrolyte), and 2 wt% NaFSI and 2 wt% NaTFSI was further introduced to obtain the ‘NaFSI and NaTFSI’ electrolyte. We found that the Na||Al cells with these two electrolytes exhibited highly consistent Na plating/stripping curves at the 40th and 70th cycles at 1 mA cm^−2^ and 1 mAh cm^−2^ (Fig. 3b, c). In addition, we observed highly consistent average Coulombic efficiencies (CEs) of 87.42% ± 1.5% and 87.47% ± 2.5% over 90 cycles in Na||Al cells using ‘no additive’ and ‘NaFSI and NaTFSI’ electrolytes, respectively, based on three cells in each group for strict statistical analysis (Fig. 3d–f). These results indicate that these F-containing additives have a minimal impact on Na plating/stripping reversibility.

In addition, the concentrations of F^−^ were obviously lower than those of Cl^−^ on the Na anode throughout cell cycling (Fig. 3g), as confirmed by time-of-flight secondary ion mass spectrometry (TOF-SIMS) (Fig. 3h). Notably, quantitative analysis showed a higher signal of NaCl_2_^−^ which is four orders of magnitude higher than that of NaF_2_^−^, indicating that NaCl is a dominant component in SEI, rather than NaF as is considered by conventional wisdom (Fig. S2a). High-resolution cryogenic transmission electron microscopy (Cryo-TEM) and scanning transmission electron microscopy (STEM) further confirmed the formation of substantial NaCl on the Na metal anode (Fig. 3i and Fig. S2b).

**Figure 4.**
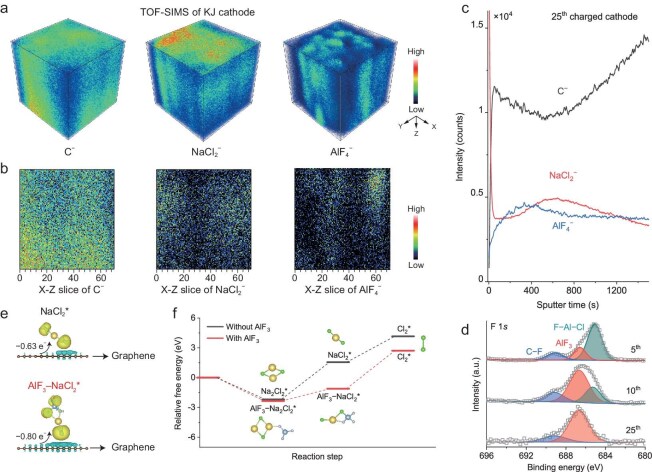
Distribution and catalytic mechanism of AlF_3_ on the cathode. (a, b) Three-dimensional and corresponding cross-section distributions of C^−^, NaCl_2_^−^, and AlF_4_^−^ secondary ion fragments constructed from TOF-SIMS depth scan on a fully charged cathode at the 25th cycle, respectively. (c) TOF-SIMS depth profiles of the secondary ion fragments of the fully charged cathode at the 25th cycle. The charge capacity and current density are 1000 mAh g^−1^ and 1 A g^−1^, respectively. (d) High-resolution F 1 *s* XPS spectra of the charged cathodes at the 5th, 10th, and 25th cycles. The charge capacity and current density are 1000 mAh g^−1^ and 1 A g^−1^, respectively. (e) Differential charge density distributions of the adsorbed reaction intermediates on the graphene without (top) and with AlF_3_ (bottom). The yellow and cyan areas represent the accumulation and depletion of electron densities. (f) Calculated Gibbs free energies of the NaCl oxidation reaction on the graphene with and without AlF_3_.

If F-containing additives cannot enhance the reversibility of the anode, would they regulate the NaCl/Cl_2_ redox reaction at the cathode? On a fully charged carbon cathode at the 25th cycle, we observed AlF_4_^−^ signals in TOF-SIMS profiles, corresponding to AlF_3_ formed at the cathode, which may be created from the AlCl_3_F⁻ anions in the electrolyte (Fig. 4a). We also observed notably different depth distributions of NaCl_2_^−^ and AlF_4_^−^ at the cathode. Specifically, NaCl_2_^−^ was mainly detected on the cathode surface, while AlF_4_^−^ showed little overlap with NaCl_2_^−^, suggesting that the NaCl crystals near AlF_3_ were oxidized due to the catalytic effect of AlF_3_ (Fig. 4b, c). X-ray photoelectron spectroscopy (XPS) confirmed the conversion of AlCl_3_F⁻ to AlF_3_ during the 5th to 25th cycles (Fig. 4d), indicating the Cl−F exchange process in AlCl_3_F⁻ anions [19]. X-ray diffraction (XRD) further identified the presence of AlF_3_ at the cathode at the 25th cycle under calcination in argon to enhance its crystallinity (Fig. S3) [20,21]. Differential charge density and Bader charge analyses indicate that the formed AlF_3_–NaCl_2_* complex could facilitate electron transfer between the carbon cathode and NaCl, contributing to efficient cleavage of the Na–Cl bond (Fig. 4e). In addition, DFT calculations confirmed that the presence of AlF_3_ could reduce the energy barrier of Na_2_Cl_2_* oxidation on graphene from 3.746 to 1.242 eV, resulting in a thermodynamically favorable oxidation of NaCl to Cl_2_ (Fig. 4f and Fig. S4). These results reveal that the F-containing electrolyte additives can yield AlF_3_ on the cathode, and further promote the oxidation kinetics of NaCl to facilitate Cl_2_ formation.

**Figure 5.**
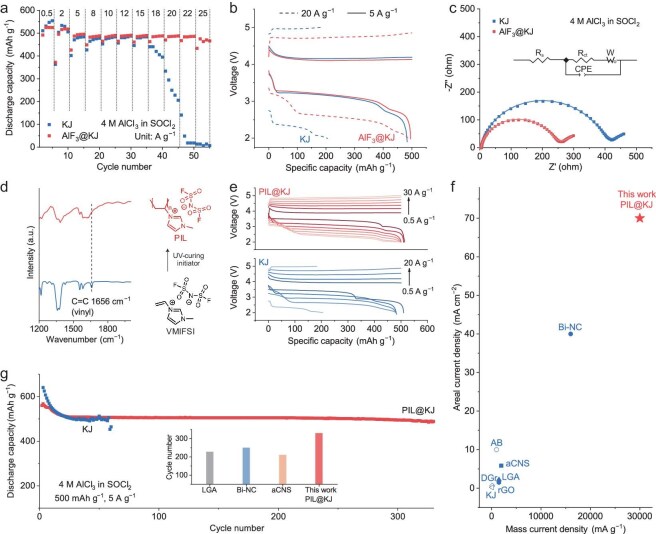
Improved rate and cycling performance *via* direct catalyst incorporation on the cathode. (a) Rate performance of Na-Cl_2_ batteries using KJ and AlF_3_@KJ cathodes with a 4 M AlCl_3_ in SOCl_2_ electrolyte. (b) Galvanostatic charge-discharge curves of KJ and AlF_3_@KJ cathodes at current densities of 5 A g^−1^ (11.7 mA cm^−2^) and 20 A g^−1^ (46.8 mA cm^−2^). (c) Nyquist plots of the 10th discharged Na-Cl_2_ batteries using KJ and AlF_3_@KJ cathodes with a 4 M AlCl_3_ in SOCl_2_ electrolyte. The inset shows the equivalent circuit used for EIS fitting. R_s_, R_ct_, CPE, and W represent contact resistance, charge-transfer resistance, constant phase element from charge transfer, and Warburg ion-diffusion resistance, respectively. (d) Schematic illustration of PIL synthesis and the corresponding FT-IR spectra. (e) Galvanostatic charge-discharge curves of Na-Cl_2_ batteries using KJ and PIL@KJ cathodes with a 4 M AlCl_3_ in SOCl_2_ electrolyte at increasing current densities from 0.5 to 30 A g^−1^ (1.2 to 70.1 mA cm^−2^). (f) Comparison of areal and mass current densities of our Na-Cl_2_ battery and state-of-the-art rechargeable Na-Cl_2_ and Li-Cl_2_ batteries [8,10,25–29]. The hollow and solid symbols represent Li-Cl_2_ and Na-Cl_2_ batteries, respectively. (g) Cycling performance of Na-Cl_2_ batteries using KJ and PIL@KJ cathodes with a 4 M AlCl_3_ in SOCl_2_ electrolyte. The charge capacity and current density are 500 mAh g^−1^ (1.2 mAh cm^−2^) and 5 A g^−1^ (11.7 mA cm^−2^), respectively. The inset shows the comparison of cycle number of our Na-Cl_2_ battery and state-of-the-art Na-Cl_2_ batteries based on different cathode materials. [8,10,28].

These new findings enable the development of new materials and incorporation strategies to improve the electrochemical performance of rechargeable Na-Cl_2_ batteries. For instance, we incorporated AlF_3_ powder directly into a KJ cathode (denoted as AlF_3_@KJ cathode). An optimal AlF_3_ concentration of 10 wt% was obtained in rate performance tests (Fig. S5). The Na-Cl_2_ battery using the AlF_3_@KJ cathode and no additive electrolyte delivered a discharge capacity of 488 mAh g⁻^1^ (calculated based on the mass of carbon unless otherwise specified, equal to 1.1 mAh cm⁻^2^) at a high current density of 20 A g^−1^ (46.8 mA cm⁻^2^), compared to only 205 mAh g⁻^1^ (0.5 mAh cm⁻^2^) for the battery with a bare KJ cathode (Fig. 5a). The AlF_3_@KJ cathode afforded a maximum current density of 25 A g⁻^1^ (58.4 mA cm⁻^2^) with a discharge capacity of 475 mAh g^−1^, surpassing 15 A g^−1^ (35.1 mA cm⁻^2^) for the bare KJ cathode (Fig. 5a). The impressive rate capability of the AlF_3_@KJ cathode can be attributed to the facilitated reaction kinetics of the NaCl/Cl_2_ redox chemistry, as evidenced by a lower charge-discharge overpotential in galvanostatic charge-discharge curves (Fig. 5b). In addition, large NaCl crystals were observed on the cathode surface when no F-based additives were present in the electrolyte, in contrast to the smaller NaCl particles observed on the cathode with the presence of those additives (Fig. S6). Nyquist plots indicated a reduced charge transfer resistance with the presence of AlF_3_ (Fig. 5c, Table S1) [22,23], which was consistent with the results in DFT calculations (Fig. 4e, f).

Despite decent electrochemical performance through the direct incorporation of AlF_3_ catalyst at the cathode, the inferior film-forming property of AlF_3_ remains a bottleneck for further performance improvement. Therefore, we synthesized a polymerized ionic liquid (PIL) with FSI⁻ anions through the photopolymerization of 1-vinyl-3-methylimidazolium bis(fluorosulfonyl)imide monomers (Fig. 5d and Fig. S7) [24]. With uniform incorporation of PIL within the KJ cathode, the PIL@KJ cathode can effectively regulate the deposition morphology of NaCl at the carbon cathode, while a highly compact NaCl layer was observed on the bare KJ cathode (Fig. S8), leading to blocked active sites and sluggish ion/electron transport. Even at a high current density of 10 A g⁻^1^ after 20 cycles, the PIL@KJ cathode maintained its porous structure, while the bare KJ cathode showed a dense passivation layer which hindered efficient ion and electron transport at the cathode (Fig. S9). In addition, the excellent film-forming property of PIL can further benefit uniform AlF_3_ distribution at the cathode, as proved by Auger electron spectroscopy (Figs S10 and S11). Therefore, a maximum current density of 30 A g^−1^ (70.1 mA cm⁻^2^) was achieved by the PIL@KJ cathode with an average CE of ∼97%, in contrast to negligible capacity and plateau at only 20 A g^−1^ (46.8 mA cm⁻^2^) for the bare KJ cathode (Fig. 5e). The achieved mass and areal current densities are highly advantageous compared to those of state-of-the-art rechargeable Na-Cl_2_ and Li-Cl_2_ batteries (Fig. 5f) [8,10,25–29]. Moreover, the PIL@KJ cathode demonstrated an improved cycle life of more than 300 cycles, surpassing only 60 cycles for the KJ cathode (Figs 5g and S12), which is also highly competitive among current rechargeable Na-Cl_2_ batteries based on advanced cathode materials [8,10,28]. The CEs exceeding 100% in the initial cycles can be attributed to additional SOCl_2_ reduction, which is likely triggered by re-activation of the carbon cathode during the oxidation of NaCl during charging. When the cathode is fully activated with stable NaCl passivation, the reduction of SOCl_2_ is suppressed with the CEs stabilized at ∼100%.

Under a lean-electrolyte condition with 20 μL electrolyte, the resulting cell based on the PIL@KJ cathode could cycle for 17 cycles, but the cell based on the KJ cathode failed to deliver stable capacities after the first cycle (Fig. S13). We further evaluated the effectiveness of both AlF_3_@KJ and PIL@KJ cathodes in rechargeable Li-Cl_2_ batteries (Fig. S14). At a high current density of 22 A g^−1^, the AlF_3_@KJ and PIL@KJ cathodes enabled higher capacities of 391 mAh g^−1^ and 464 mAh g^−1^, respectively, surpassing 179 mAh g^−1^ for the KJ cathode (Fig. S14a). Even at a harsh current density of 28 A g^−1^, the PIL@KJ cathode showed a discharge capacity of 429 mAh g^−1^. Galvanostatic charge-discharge curves of these batteries at 10 A g^−1^ showed the lowest overpotential of 1.21 V for the PIL@KJ cathode (Fig. S14b). These results have validated the applicability of the proposed mechanism in rechargeable Li-Cl_2_ batteries.

The clarified working mechanism of the F-containing electrolyte additives can further unlock new cathode and electrolyte materials for high-performance rechargeable Na-Cl_2_ batteries. For instance, we synthesized AlCl_x_F_y_ as an effective electrolyte additive, which shows enhanced compatibility and dispersibility in chloroaluminate electrolyte due to the presence of Cl ions, and improved catalytic activity (Lewis acidity) based on F doping (see Methods and Fig. S15a, b) [30,31]. The addition of AlCl_x_F_y_ at an ultralow content of 0.5 wt% into the basic electrolyte can improve the cycling stability of rechargeable Na-Cl_2_ batteries (Fig. S15c, d). More importantly, it can effectively extend the electrolyte system of rechargeable Na-Cl_2_ batteries, e.g. the GaCl_3_−SOCl_2_ system (Fig. S16). These results can enrich the choices of electrolyte and cathode materials for rechargeable Na-Cl_2_ batteries, thereby promoting their real-world applications. More broadly, the ‘anode additive-cathode catalyst’ strategy demonstrated in this work could inspire new approaches to improve the electrochemical performance of other rechargeable batteries, such as sulfur and oxygen systems, by transforming traditional anode-protective additives into efficient cathode catalysts.

## CONCLUSION

In summary, we have elucidated the distinct mechanism of F-containing electrolyte additives in rechargeable Na-Cl_2_ batteries, which is fundamentally different from that observed in conventional Li- and Na-metal batteries. We validate that a spontaneous chemical reaction occurs between the F-containing additives and AlCl_3_ during electrolyte preparation, leading to the formation of AlF_3_ at the cathode, which can reduce the energy barrier of NaCl oxidation and facilitate the charge transfer between the carbon cathode and NaCl, thereby enhancing both rate capability and cycling stability. Building on these insights, we developed a polymerized ionic liquid based on FSI⁻ anions, designed for improved film-forming ability and chemical compatibility. This tailored catalyst enables impressive rate capability (30 A g^−1^) and cycling stability (more than 300 cycles) in rechargeable Na-Cl_2_ batteries. Our results not only clarify the working mechanism of F-containing electrolyte additives in rechargeable Na-Cl_2_ batteries but, in a broader context, open up a new avenue for the rational design of advanced electrode and electrolyte systems for practical alkali metal-Cl_2_ batteries.

## Supplementary Material

nwaf333_Supplemental_Files
