# Supplementary material for: Unveiling cathode catalysis of fluorinated electrolyte additives for high-performance Na-Cl2 batteries
Source: Natl Sci Rev. 2025 Aug 12;12(10):nwaf333. doi: 10.1093/nsr/nwaf333 (PMC12491998; doi:10.1093/nsr/nwaf333)
Supplement: nwaf333_Supplemental_Files [file nwaf333_supplemental_files.zip › Supplementary data.pdf]

Supplementary Information

Unveiling cathode catalysis of fluorinated electrolyte additives for high-performance Na-Cl_2 batteries

Qiuchen Xu[a]+, Shanshan Tang[a]+, Shuo Wang[a], Anrong Chen[a], Yan Wang[a], Shitao Geng[a], Bin Yuan[a], Chengxiao Zhang[a], Qianyun Chen[a], Zhaofeng Ouyang[a], Feng Zhu[a], Xiaoju Zhao[a]* and Hao Sun[a]*

[a]Frontiers Science Center for Transformative Molecules, School of Chemistry and Chemical Engineering, Zhangjiang Institute for Advanced Study, Shanghai Jiao Tong University, Shanghai 200240, China.

+These authors contributed equally to this work.

This file includes:

Materials and Methods (Pages S2-S7)
Figure S1 to S16 (Pages S8-S23)
Table S1 (Page S24)
Supplementary References (Page S25)

1. Materials and Methods

1.1 Materials: Thionyl chloride ($SOCl_2$, 99.0%) was obtained from Energy Chemical (Shanghai). Aluminium chloride ($AlCl_3$, 99.0%) was purchased from TCI Development Co., Ltd. Sodium bis(fluorosulfonyl)imide (NaFSI, 99.9%) and sodium bis(trifluoromethylsulfonyl)imide (NaTFSI, 99.9%) were sourced from Changde Dado New Material Co., Ltd. Gallium chloride ($GaCl_3$, 99.99%), aluminum fluoride (AlF_3, 99.99%), 2-hydroxy-2-methylpropiophenone (HMPP, 98.0%) and N-methylpyrrolidone (NMP, 99.5%, safedry) were purchased from Adamas Reagent (Shanghai). 1-vinyl-3-methylimidazolium bis(fluorosulfonyl)imide (VMI-FSI, 99.9%) was purchased from Shanghai Fujie Chemical Co., Ltd. Sodium metal cubes (Na, 99.7%) and trichlorofluoromethane ($CFCl_3$, 99.8%) were acquired from Shanghai Aladdin Biochemical Technology Co., Ltd. Nickle foam (Ni, 0.5 mm thickness), ketjenblack (KJ, ECP-600JD), and polytetrafluoroethylene emulsion binder (PTFE, 60 wt% aqueous dispersion, Daikin, D-210C) were purchased from Cyber Electrochemical Materials. Li foils (thickness of 500 μm, 99.95%) and carbon-coated aluminum foils were obtained from Guangdong Canrd New Energy Technology Co., Ltd. Prior to use, NaFSI and NaTFSI were dried in a vacuum oven at 90 °C overnight. All the other chemicals were used as received without further purification.

1.2 Preparation of $AlCl_xF_y$: $AlCl_xF_y$ was synthesized according to a previous literature.[1] Specifically, 10 g of $AlCl_3$ was placed in a round-bottomed flask equipped with a condenser and cooled to −78 °C using a liquid nitrogen/ethyl acetate bath. 45 mL of $CFCl_3$ was then slowly dropwise added to the flask over 15 min under N_2 atmosphere. The resulting suspension was first stirred at −78 °C for 1 h, and then stirred at room temperature for another 3 h. The exothermic reaction maintained solvent reflux (b.p. 23.8 °C) at room temperature. After removal of the solvent under vacuum, yellow $AlCl_xF_y$ powder was obtained and stored in an argon-filled glovebox. Wavelength dispersive X-ray fluorescence spectrometry confirmed a molar ratio of 0.12:2.88 for Cl: F in the synthesized $AlCl_xF_y$.

1.3 Preparation of polymerized ionic liquid (PIL): PIL was synthesized according to a previous literature,[2] carried out in an argon-filled glove box with the contents of H_2O and O_2 below 1 ppm. Specifically, 1 g of VMI-FSI monomer and 0.016 g of HMPP initiator were mixed and stirred at 60 °C for 2 h. The mixture was then subjected to UV-induced polymerization (λ = 455 nm) for 3 h to obtain the PIL product.

1.4 Preparation of the electrolytes: All electrolytes were prepared in an argon-filled glove box with the contents of H_2O and O_2 below 1 ppm. The $SOCl_2$-based electrolyte

was prepared by dissolving 4 M $AlCl_3$ in $SOCl_2$, followed by adding NaFSI and NaTFSI (both 2 wt% of the total weight of $AlCl_3$ and $SOCl_2$). The mixture was stirred at 800 rpm for 4 h to obtain the 'NaFSI and NaTFSI' electrolyte. For comparison, the 'no additive' electrolyte was prepared by adding 4 M $AlCl_3$ to $SOCl_2$, and stirred at 800 rpm for 30 min.

***1.5 Preparation of the electrodes*:** To prepare the cathode slurry, KJ and PTFE were mixed in ethanol at a weight ratio of 9:1. The mixture was then sonicated until the carbon materials were uniformly dispersed. Ni foam with a diameter of 14 mm was fabricated using a manual disk cutter (MTI, MSK-T-10), and cleaned in ethanol *via* ultrasonication for 30 min. The uniformly dispersed cathode slurry was slowly dropped (150 μL each time) onto the Ni foam hovered over an 80 °C hot plate, allowing ~5 min between each addition to ensure complete ethanol evaporation. This process was repeated until the carbon mass loading reached 2-3 mg cm^{-2}. The resulting carbon cathodes were dried overnight in a vacuum oven at 80 °C, and then roll-pressed into thinner electrodes. AlF_3@KJ and PIL@KJ cathodes were prepared using the same method, with the initial slurry composition modified to KJ: AlF_3/PIL: PTFE = 9:1:1. To prepare the carbon cathode for lean-electrolyte condition, the KJ cathodes were prepared by mixing KJ and PVDF in NMP with a weight ratio of 9:1, followed by grounding using an agate mortar for 2 h at 25 ºC. The slurry was then coated onto a carbon-coated aluminum foil and dried under vacuum at 100 °C for 12 h, which was further rolled and pressed using a stainless-steel cylinder under a pressure of 6 MPa to obtain KJ cathodes. PIL@KJ cathodes were prepared using the same method, with the initial slurry composition modified to KJ: AlF_3/PIL: PVDF = 9:1:1.

***1.6 Preparation and characterization of the rechargeable Na-Cl_2 batteries*:** All batteries were assembled inside an argon-filled glove box with the contents of H_2O and O_2 below 1 ppm. The Na metal anodes were prepared by removing the surface oxide layer from a rinsed Na metal cube using a clean blade, followed by pressing the Na metal into a thin foil with a diameter of 14 mm. For making Na-Cl_2 coin cells (2032 type, grade 316 stainless steel), the glass fibre separator (GF/D, Whatman) was cut into 16 mm-diameter discs and vacuum-dried overnight at 120 °C. A piece of KJ-based cathode (diameter of 14 mm) and a Na foil (diameter of 14 mm) were separated by the glass fibre separator with 150 μL electrolyte. For making Na-Cl_2 coin cells under lean-electrolyte conditions, the ceramic-coated polypropylene separator (PP/Al_2O_3) was cut into 16 mm-diameter discs. A piece of KJ-based cathode (diameter of 14 mm) and a Na foil (diameter of 14 mm) were separated by the separator with 20 μL electrolyte. For preparing Na-Al coin cells, a piece of Na foil (diameter of 14 mm) was paired with an Al foil (diameter of 14 mm) separated by a GF/D separator with 120 μL electrolyte. All

coin cells were hermetically sealed using a digital pressure controllable electric crimper (MTI, MSK-160E). Galvanostatic charge-discharge measurements were conducted on a Neware battery testing system (CT-4008Tn-5V6A-S1). All the electrochemical measurements were performed at 25 °C in a thermostatic test chamber (Neware MHW-200). The rate and cycling performance of the Na-Cl_2 coin cells were tested at a charge capacity of 500 mAh g^{-1} with a discharge cut-off voltage of 2.0 V. The cycling performance of the Na-Al coin cells was tested between −2.0 and 2.0 V. The Coulombic efficiency (CE) is calculated as the ratio of discharge capacity divided by the charge capacity in the preceding charge cycle. Electrochemical impedance spectroscopy (EIS) measurements were conducted on a CHI660E electrochemical work station. Three-electrode electrochemical impedance tests were performed on CR2032 coin cells with a Na metal foil serving as both counter and reference electrodes, and the carbon cathode as the working electrode. Prior to the EIS measurement, the cell was retained at the open-circuit voltage (OCV) condition for 30 minutes for stabilization. The frequency range spanned from 0.1 to 10^5 Hz with an amplitude of 5 mV and 12 data points were collected per decade of frequency.

1.7 Characterizations

***1.7.1 Scanning electron microscopy (SEM)*:** SEM was conducted on a field emission scanning electron microscopy (ZEISS, Gemini300) at an accelerating voltage of 5 kV. The cathodes were rinsed with $SOCl_2$ for three times to remove any residual electrolyte, followed by vacuum treatment to remove $SOCl_2$.

***1.7.2 Transmission electron microscopy (TEM)*:** Cryo-TEM (200 kV) was performed using an FEI Talos F200X G2 microscope with liquid nitrogen cooling. For cryo-TEM sample preparation, a Na/Au grid coin cell was assembled to deposit Na metal at a deposition capacity of 0.02 mAh cm^{-2} and a current density of 0.2 mA cm^{-2}. The coin cell was then disassembled inside an argon-filled glove box, and the Na-deposited Au grid was rinsed with diglyme for three times to remove residual electrolyte, and dried under vacuum to remove diglyme. It was then transferred to a cryo-holder (Fischione 2550) in the glove box without exposure to air, and the cryo-holder was placed in the microscope for characterization. Liquid nitrogen was used to keep the cryo-holder at a stable temperature of approximately −170 °C during analysis.

***1.7.3 X-ray Diffraction (XRD) patterns*:** XRD patterns were collected on a Rigaku X-ray diffractometer ARL Equinox at a rate of 2° min^{-1} over the range of 20–90° (2θ). To prevent air-sensitive sample degradation, all samples were carefully loaded and

hermetically sealed in an airtight sample holder under inert atmosphere prior to transfer for XRD characterization.

1.7.4 X-ray photoelectron spectroscopy (XPS)**:** XPS analysis was conducted on a Thermo ESCALAB 250Xl with a monochromatic Al Kα source (hv = 1486.6 eV). The XPS samples were disassembled in the glove box. The cathodes were rinsed with $SOCl_2$ to remove any residual electrolyte, followed by vacuum treatment to eliminate the solvents. These samples were then transferred into an Ar-filled chamber for XPS measurement without exposure to air. The vacuum level of the analysis chamber was maintained below 5×10^{-9} Torr. The test area was a circle with a diameter of 500 μm and all binding energies were calibrated with C 1s peak (284.8 eV).

1.7.5 Auger electron spectroscopy (AES)**:** The AES characterization was conducted using a PHI 710 scanning Auger nanoprobe, equipped with a thermally assisted Schottky field-emission electron gun and a coaxial cylindrical mirror analyzer. SEM and AES measurements were performed with an accelerating voltage of 10 kV, a current of 10 nA, and a sample tilting angle of 45° from the surface normal to the electron gun. AES depth profiling was performed by argon-ion sputtering at 2 keV and scanning the ion beam over a 2 × 2 mm^2 area.

1.7.6 Time-of-flight secondary-ion mass spectrometry (TOF-SIMS)**:** Cathode and anode samples were extracted from cycled cells in an argon-filled glovebox, rinsed with $SOCl_2$ and diglyme, respectively, vacuum-dried in the glovebox antechamber overnight. TOF-SIMS measurements were performed using an ION-TOF TOF-SIMS 5 instrument with the analysis chamber maintained at $<1.1 \times 10^{-9}$ mbar. Depth profiling was performed using a Bi^{3+} ion beam at 60 keV in delay extraction mode for organic imaging, with both primary and sputter ion beams incident at a 45° angle relative to the sample surface. For the cathode, a Cs^+ sputter beam at 2 keV was used with a raster size of 280 × 280 μm^2, while the analysis area was 70 × 70 μm^2. For the anode, a Cs^+ sputter beam at 500 eV was used with a raster size of 200 × 200 μm^2, while the analysis area was 50 × 50 μm^2.

1.7.7 High-resolution mass spectra (HRMS): HRMS were conducted using a Waters Micro mass quadrupole/time-of-flight (Q-ToF) Premier mass spectrometer (G2-XS/APGC, 50-2000 m/z) in a negative mode. The solvent system was acetonitrile with a flow rate of 0.1 mL min^{-1}. The data was processed using MassLynx V4.1 software.

1.7.8 Nuclear Magnetic Resonance (NMR)**:** The liquid NMR experiments were performed at 25 °C on a Bruker Avance NEO 500 MHz spectrometer equipped with an

iProbe. Prior to measurements, NMR tubes (5 mm high throughput) were dried under vacuum at 80 °C for 12 h. In an argon-filled glovebox, 400 μL of electrolyte was loaded into the NMR tube. A coaxial insert that contains 100 μL of deuterated solvent (DMSO-d_6) was inserted into the NMR tube to ensure the analysis while preserving the original electrolyte. ^{27}Al NMR spectra were acquired with a spectral width of 1,009.6 ppm, an acquisition time of 0.25 s, and 32 number of scans. ^{19}F NMR spectra were acquired with a spectral width of 590.2 ppm, an acquisition time of 0.24 s, and 128 number of scans. Solid-state NMR experiment was performed at 25 °C on a Bruker Avance NEO 600 MHz spectrometer using magic-angle spinning (MAS) probes and a single-pulse excitation sequence. ^{19}F NMR spectrum was acquired with a 1.9 mm probe spinning at 40 kHz. ^{27}Al NMR spectrum was acquired with a 3.2 mm probe spinning at 20 kHz.

***1.7.9 Ion chromatography*:** Ion chromatography analysis was performed using a Metrohm 930 system equipped with a Metrosep A Supp 5-250/4.0 anion-exchange column (250 mm × 4.0 mm). Anodes were disassembled inside an argon-filled glovebox, thoroughly washed three times with diglyme to remove residual electrolytes, then vacuum-dried for 8 h. Subsequently, 2 mL of ultrapure water was added to the dried sample to dissolve ionic species. The resulting solution was filtered through a 0.22 μm membrane filter to remove particulate matter. The eluent consisted of 3.2 mM Na_2CO_3 and 1.0 mM $NaHCO_3$.

***1.7.10 Gel permeation chromatography (GPC)*:** GPC analyses were carried out on Agilent 1260 Infinity II (PL GPC 50, America). The measurements used *N,N*-dimethylformamide as an eluent at a flow rate of 1 mL min^{-1} at 40 ºC. The sample concentrations were 1 mg mL^{-1} and the injection volumes were 1,000 μL. Polystyrene standards (PSS Polymer Standards Service Inc., Germany) with narrow molecular weight distributions were used as calibration (calibration range: 300–67,500 g mol^{-1}).

***1.7.11 Thermogravimetric Analysis (TGA)*:** Thermogravimetric analysis (TGA) experiments were performed on a Discovery TGA550 instrument at a linear heating rate of 10 °C min^{-1} from 30 to 800 °C under N_2 atmosphere.

***1.8 Theoretical calculations*:** Gibbs free energy calculations of the electrolyte reaction were conducted using the Gaussian 16 program package.[3] The geometries of all molecules under study were optimized and calculated at the BP86/TZVP level of theory.[4-6] Additionally, a dispersion correction with the Becke–Johnson damping function was applied.[7,8] Vibrational frequency analysis was carried out at the same level of theory to verify that the molecules were at the minima of their potential energy surfaces, with zero imaginary frequencies observed in all cases. Single-point energies

were also calculated using the same theoretical approach. Furthermore, the solvation effects were accounted for using the implicit Solvation Model Based on Density (SMD) [9] with quinoline as the solvent for calculation which has a similar dielectric constant with our $SOCl_2$ solvent.

NaCl oxidation reaction calculations were performed using the Vienna Ab Initio Simulation Package (VASP).[10,11] The generalized gradient approximation method with the Perdew-Burke- Ernzerhof (PBE) exchange-correlation function was used to manage the electron exchange and correlation energy.[12] The plane wave basis (kinetic energy cut-off value 450 eV) was used to describe the valence electrons. The atomic positions were fully optimized until the energy and forces converged to $1*10^{-5}$ eV and 0.03 eV $Å^{-1}$, respectively. All calculations were using the k-point sampling obtained from the gamma center with a mesh 3*3*1. The reaction energy was based as following steps:

Step1: $2NaCl + Substrate \rightarrow 2NaCl^{*} + Substrate$

Step 2: $2NaCl^{*} + Substrate \rightarrow Na^{+} + NaCl_2^{*} + Substrate$

Step 3: $NaCl_2^{*} + Substrate \rightarrow Na^{+} + Cl_2^{*} + Substrate$

$E = \sum E_{product} - \sum E_{reactant}$

The charge density differences were considered as following:

$\delta_{\rho} = \rho_{A+B} - \rho_{A} - \rho_{B}$

While ρ_{A+B} is the charge density of intermediate adsorption at electrode, ρ_{A} and ρ_{B} is the charge density of intermediate and electrode, respectively.

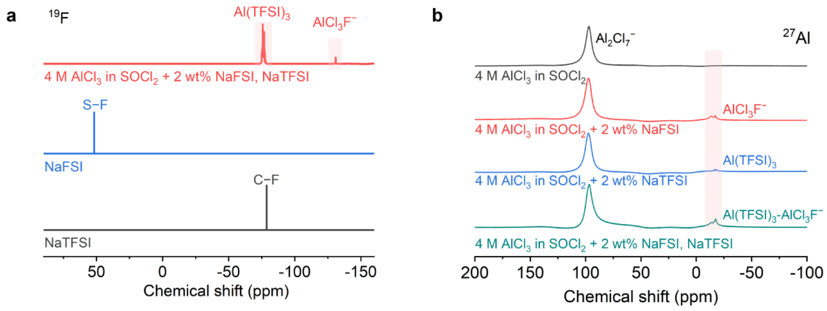

Figure S1. Spontaneous chemical reactions of F-containing additives in the electrolyte of rechargeable Na-Cl_2 batteries. (a) ^{19}F NMR spectra of NaFSI, NaTFSI, and 4 M $AlCl_3$ in $SOCl_2$ with 2 wt% NaFSI and 2 wt% NaTFSI electrolyte. The reaction of $TFSI^-$ anions in the $AlCl_3$−$SOCl_2$ electrolyte was evidenced by the multiple signals at −78 ppm, which could be attributed to the existence of various isomers in $Al(TFSI)_3$, compared to the clean singlet at −78 ppm observed in bare NaTFSI.[13] (b) ^{27}Al NMR spectra of the electrolytes comprising 4 M $AlCl_3$ in $SOCl_2$ with and without F-containing additives.

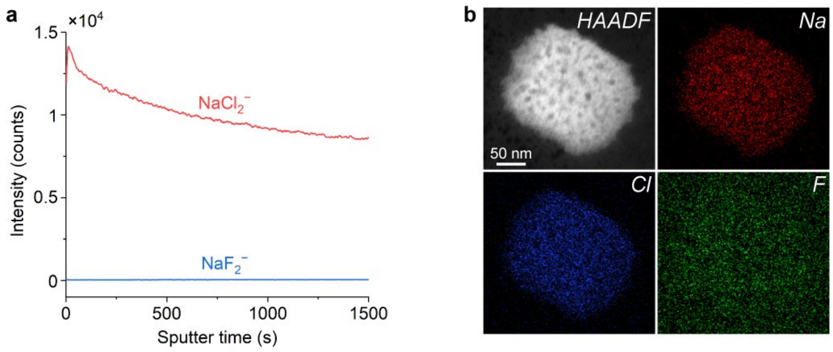

Figure S2. Impact of F-containing electrolyte additives on solid-electrolyte interphase layer. (a) TOF-SIMS depth profile of secondary ion fragments on the 25th charged anode. The charge capacity and current density are 1,000 mAh g^{-1} and 1 A g^{-1}, respectively. (b) High-angle annular dark-field (HAADF) and the corresponding element mapping images of Na-plated Au grid using scanning transmission electron microscopy (STEM). A Na/Au grid coin cell was assembled to deposit Na metal at a deposition capacity of 0.02 mAh cm^{-2} and a current density of 0.2 mA cm^{-2}.

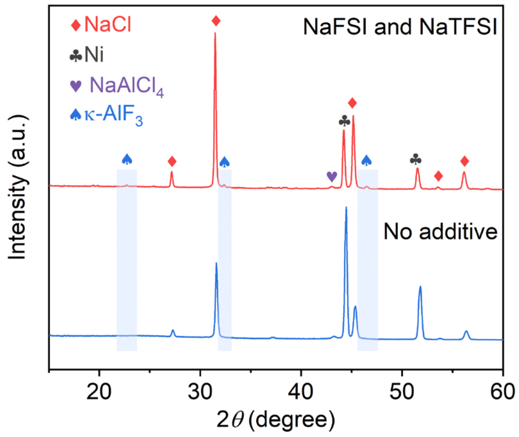

Figure S3. XRD patterns of the 25th charged cathodes using the $AlCl_3$–$SOCl_2$ electrolyte with and without F-containing additives. The charge capacity and current density are 1,000 mAh g^{-1} and 1 A g^{-1}, respectively. The cathodes were annealed in argon atmosphere at 500 °C for 2 h with a controlled heating ramp of 5 °C min^{-1} to enhance crystallinity prior to XRD characterization.

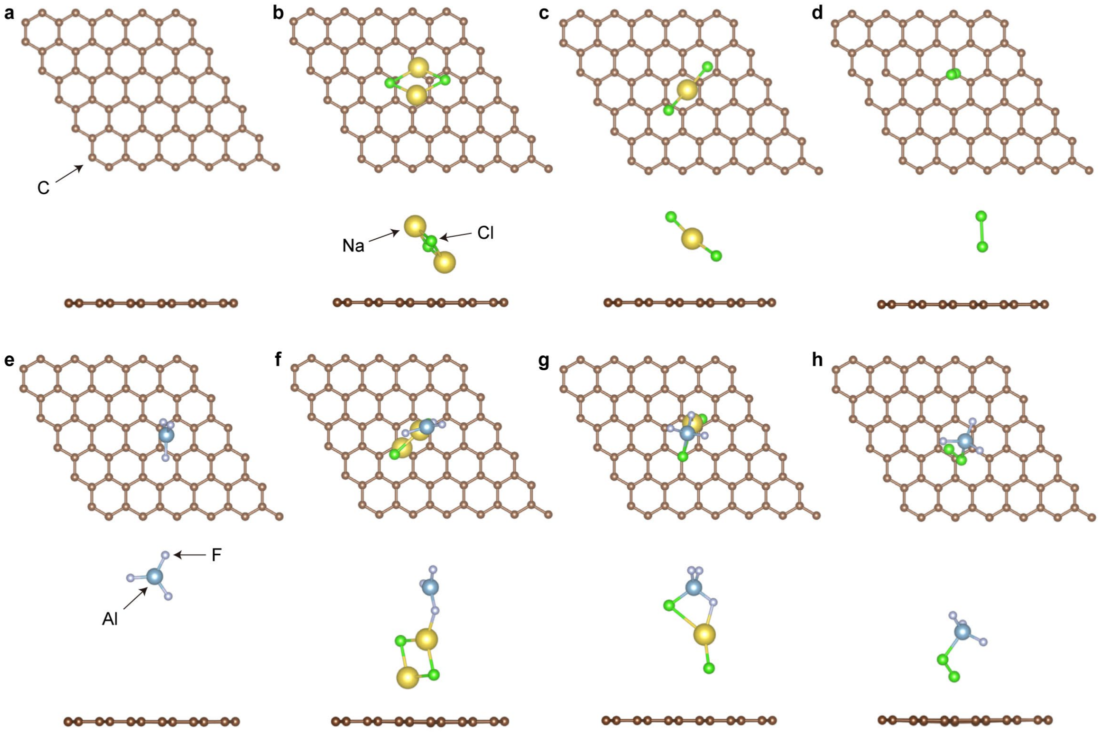

Figure S4. Optimized structures of the intermediates on bare graphene without (a-d) and with (e-h) the incorporation of the AlF_3 catalyst for the oxidation reaction of NaCl.

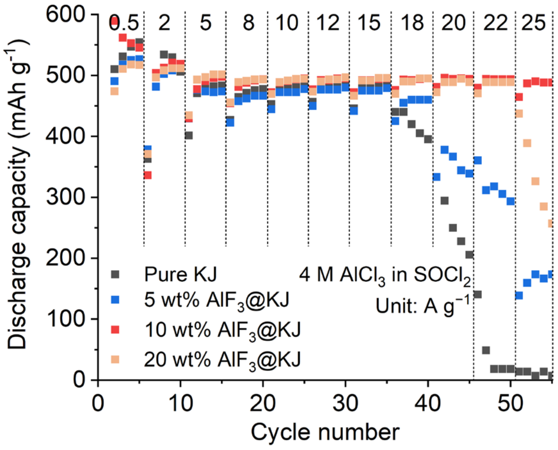

Figure S5. Rate performance of Na-Cl_2 batteries using the KJ and AlF_3@KJ cathodes with a 4 M $AlCl_3$ in $SOCl_2$ electrolyte.

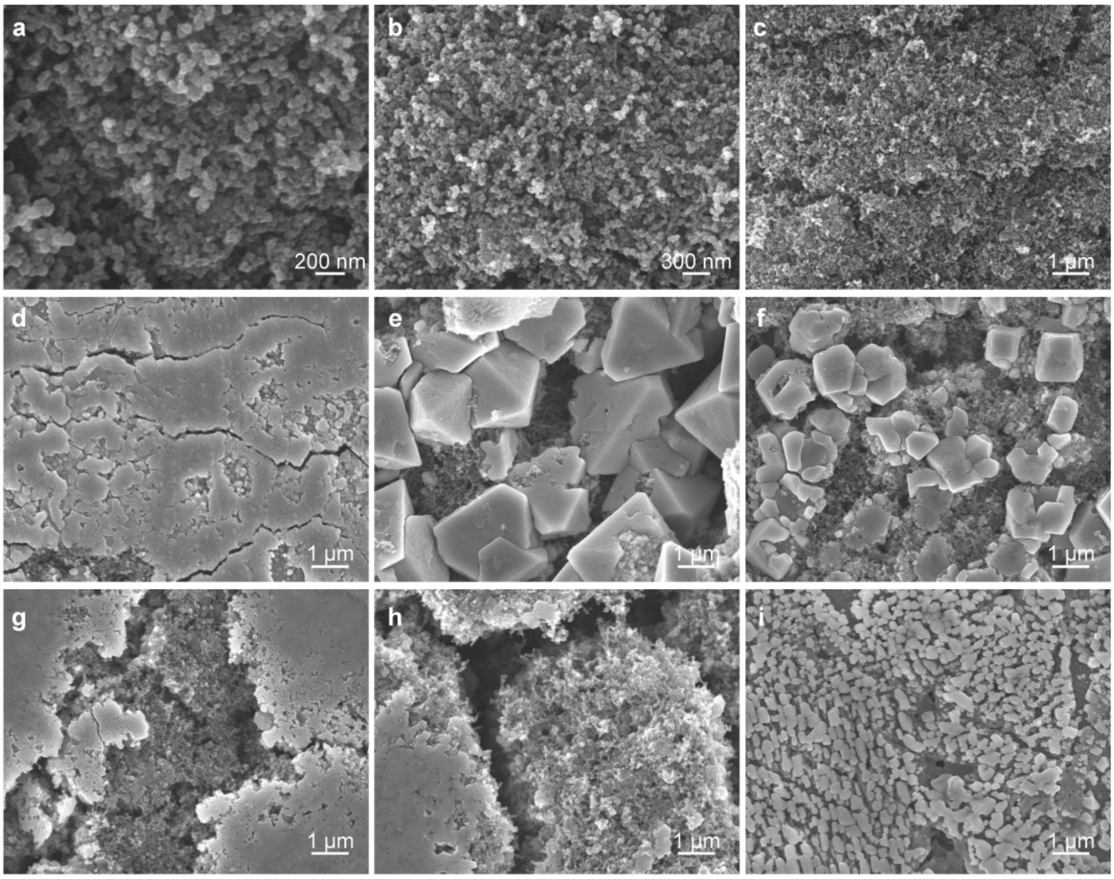

Figure S6. (a-c) SEM images of the as-prepared KJ cathode. (d-f) SEM images of a charged KJ cathode with a F-free electrolyte (4 M $AlCl_3$ in $SOCl_2$) after 20 cycles at the current densities of 1 A g^{-1} (d), 2 A g^{-1} (e), and 5 A g^{-1} (f), respectively. (g-i) SEM images of a charged KJ cathode with a F-containing electrolyte (4 M $AlCl_3$ in $SOCl_2$ with 2 wt% NaFSI and NaTFSI) after 20 cycles at the current densities of 1 A g^{-1} (g), 2 A g^{-1} (h), and 5 A g^{-1} (i), respectively. The charge capacity in all figures is 1,000 mAh g^{-1}.

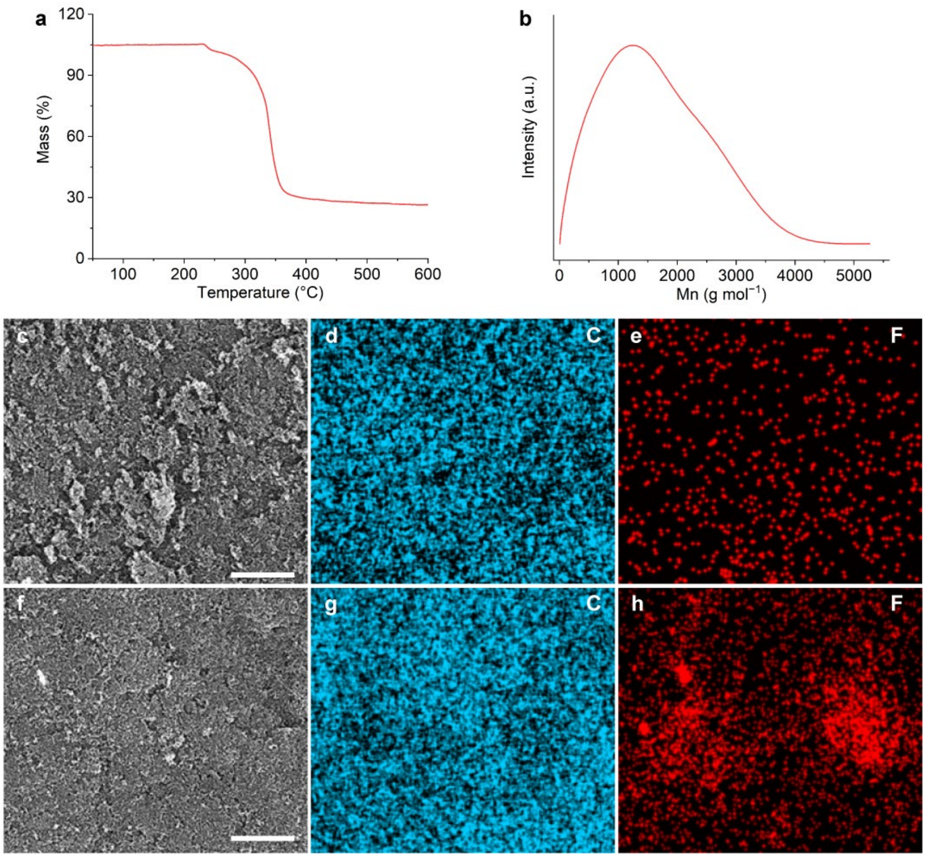

Figure S7. Characterization of the polymerized ionic liquid (PIL) catalyst and the corresponding catalyst-loaded cathode. (a) Thermo-gravimetric (TG) curve of PIL which exhibited excellent thermal stability below 200 °C. (b) Gel permeation chromatography (GPC) profile for PIL. The number-average molecular weight (M_n) of PIL was 1,110 g mol^{-1}. (c-e) SEM images of the PIL@KJ cathode and the corresponding element mapping images. Scale bar, 5 µm. (f-h) SEM image of the AlF_3@KJ cathode and the corresponding element mapping images. Scale bar, 5 µm.

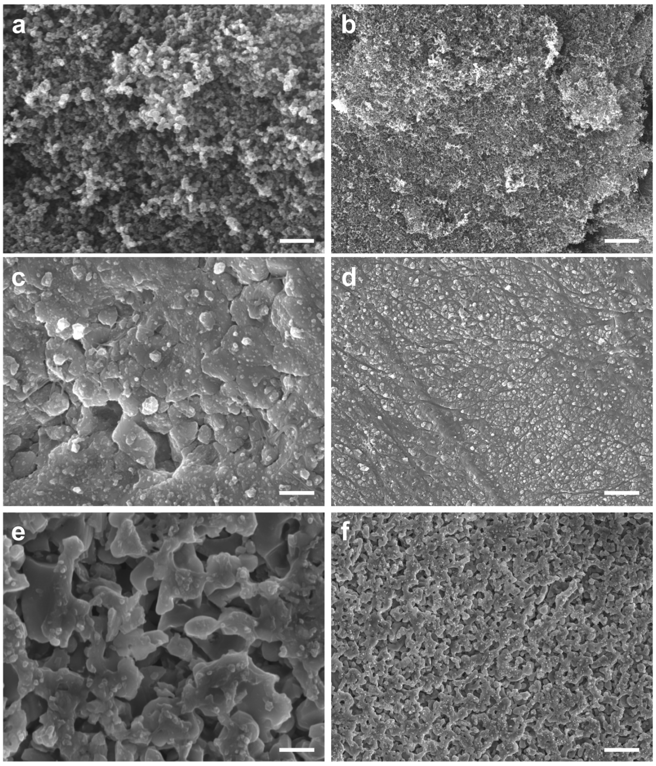

Figure S8. Morphology characterization of different cathodes after cycling. (a, b) SEM images of KJ cathode at low and high magnifications, respectively. (c, d) SEM images of a discharged KJ cathode with a F-free electrolyte (4 M $AlCl_3$ in $SOCl_2$) after 40 cycles at low and high magnifications, respectively. (e, f) SEM images of a discharged PIL@KJ cathode with a F-free electrolyte (4 M $AlCl_3$ in $SOCl_2$) after 40 cycles at low and high magnifications, respectively. Scale bars in (a), (c), and (e) are 500 nm. Scale bars in (b), (d), and (f) are 2 μm. The charge capacity and current density are 1,000 mAh g^{-1} and 1 A g^{-1}, respectively.

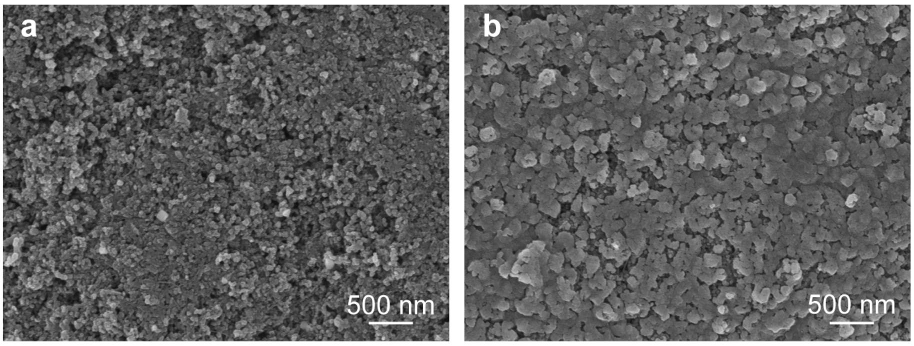

Figure S9. SEM images of charged PIL@KJ (a) and KJ (b) cathodes after 20 cycles. The charge capacity and current density are 500 mAh g^{-1} and 10 A g^{-1}, respectively.

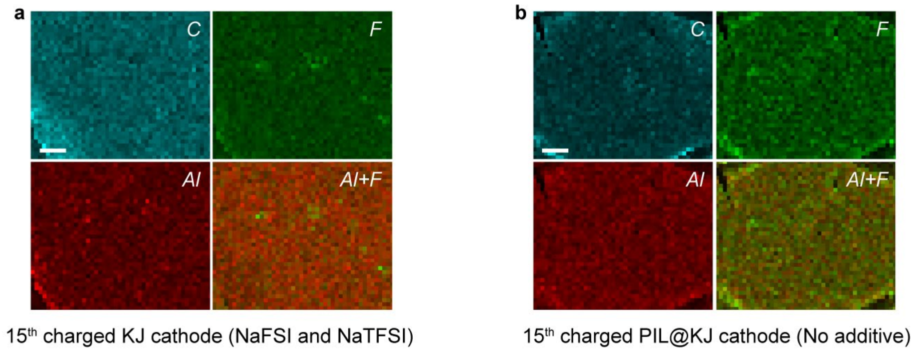

Figure S10. Auger electron spectroscopy mapping images for C, Al and F of the 15th charged KJ cathode with a F-containing electrolyte (a) and PIL@KJ cathode with a F-free electrolyte (b). The charge capacity and current density are 1,000 mAh g^{-1} and 500 mA g^{-1}, respectively. Scale bar, 10 μm.

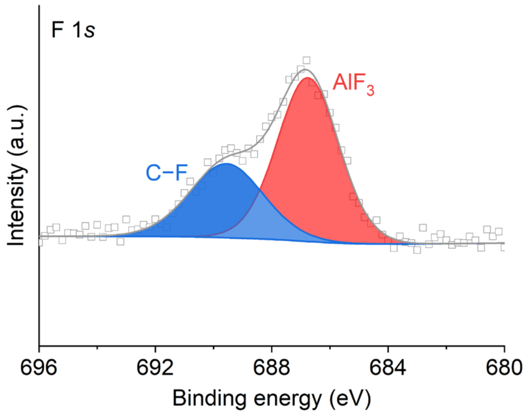

Figure S11. High-resolution F 1*s* XPS spectra of the charged PIL@KJ cathode at the 15th cycle. The charge capacity and current density are 1,000 mAh g^{-1} and 1 A g^{-1}, respectively.

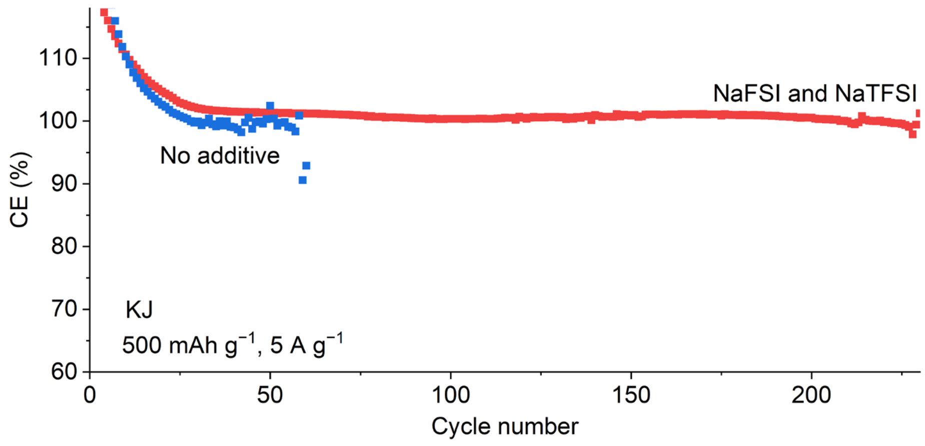

Figure S12. Cycling performance of Na-Cl_2 batteries using $AlCl_3$–$SOCl_2$ electrolyte with and without NaFSI and NaTFSI as the F-containing additives. The charge capacity and current density are 500 mAh g^{-1} and 5 A g^{-1}, respectively.

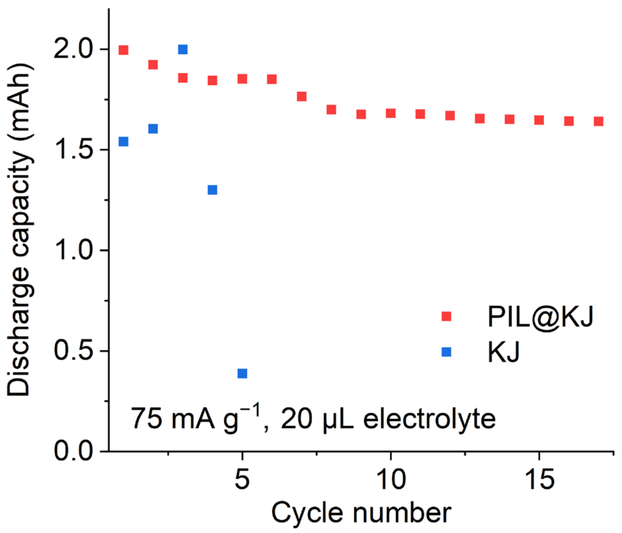

Figure S13. Cycling performance of Na-Cl_2 batteries using KJ and PIL@KJ cathodes with a 4 M $AlCl_3$ in $SOCl_2$ electrolyte. The charge capacity and current density are 1,100 mAh g^{-1} and 75 mA g^{-1}, respectively. The mass loading of KJ is 1.63 mg cm^{-2} and the electrolyte volume is 20 μL.

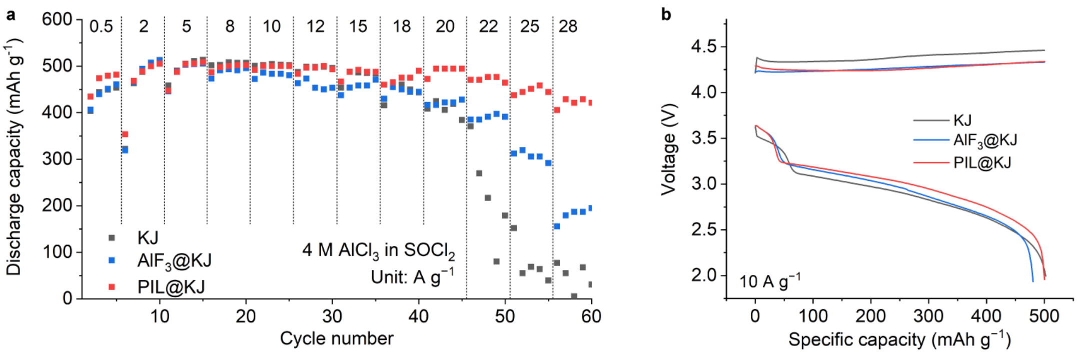

Figure S14. (a) Rate performance of Li-Cl_2 batteries using KJ, AlF_3@KJ, and PIL@KJ cathodes with a 4 M $AlCl_3$ in $SOCl_2$ electrolyte. (b) Galvanostatic charge-discharge curves of KJ, AlF_3@KJ, and PIL@KJ cathodes at a charge capacity and current density of 500 mAh g^{-1} and 10 A g^{-1}, respectively.

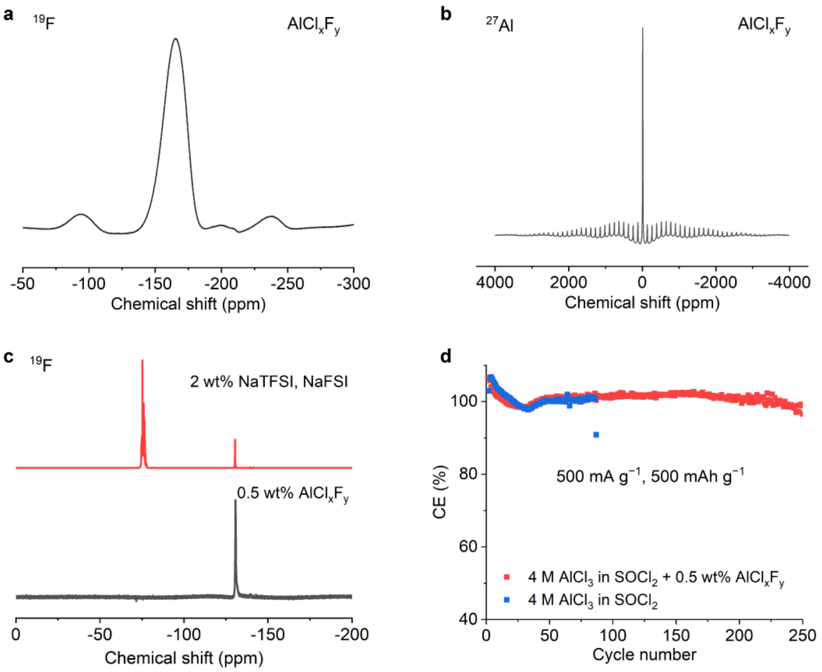

Figure S15. Characterization of $AlCl_xF_y$ and electrochemical performance of the obtained rechargeable Na-Cl_2 batteries. (a, b) ^{19}F and ^{27}Al MAS NMR spectra (600 MHz) of $AlCl_xF_y$, respectively. (c) ^{19}F NMR spectra of the 4 M $AlCl_3$ in $SOCl_2$ electrolyte with the addition of 2 wt% NaFSI and 2 wt% NaTFSI or 0.5 wt% $AlCl_xF_y$. (d) Cycling performance of the rechargeable Na-Cl_2 batteries using $AlCl_3$−$SOCl_2$ electrolyte with and without 0.5 wt% $AlCl_xF_y$ additive. The charge capacity and current density are 500 mAh g^{-1} and 500 mA g^{-1}, respectively.

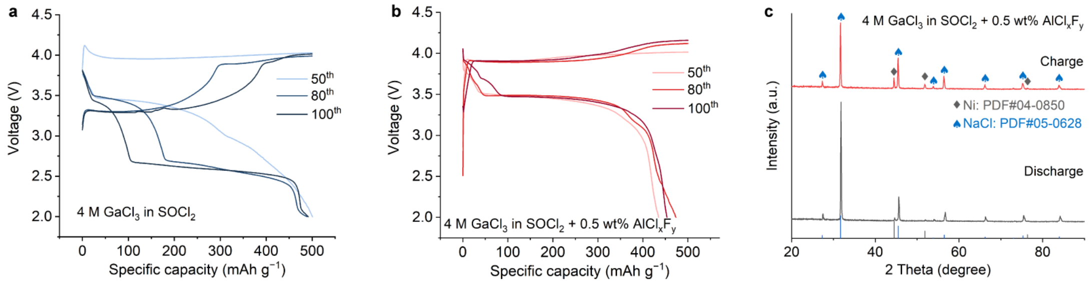

Figure S16. (a, b) Galvanostatic charge-discharge curves of the rechargeable Na-Cl_2 batteries using $GaCl_3$−$SOCl_2$ electrolytes with and without 0.5 wt% $AlCl_xF_y$ additive, respectively. The charge capacity and current density in (a, b) are 500 mAh g^{-1} and 500 mA g^{-1}, respectively. (c) XRD patterns of the cathodes using the $GaCl_3$−$SOCl_2$ electrolytes with 0.5 wt% $AlCl_xF_y$ additive in the fully discharged and charged to 1,000 mAh g^{-1} states. The current density is 200 mA g^{-1}.

Table S1. EIS fitting results for Na-Cl_2 batteries using KJ and AlF_3@KJ cathodes with a 4 M $AlCl_3$ in $SOCl_2$ electrolyte in Figure 5c.

Samples	R_s (Ω)	R_{ct} (Ω)	χ^2
KJ	5.1	360.8	0.0039
AlF_3@KJ	4.3	213.4	0.0045

R_s, and R_{ct} represent contact resistance and charge-transfer resistance, respectively. Chi-square (χ^2) tests were used to determine the statistical difference between observed and fitted values.

References

1. Krahl T, Stösser R, Kemnitz E *et al.* Structural Insights into Aluminum Chlorofluoride (ACF). *Inorg Chem* 2003; **42**: 6474-6483.
2. Qin Y, Wang H, Zhou J *et al.* Binding FSI^{-} to Construct a Self-Healing SEI Film for Li-Metal Batteries by In situ Crosslinking Vinyl Ionic Liquid. *Angew Chem Int Ed* 2024; **63**: e202402456.
3. Frisch MJ, Trucks GW, Schlegel HB, Scuseria GE, *Gaussian 16*, Revision A.03 (Gaussian, Inc, Wallingford, CT, 2016).
4. Becke AD. A new mixing of Hartree–Fock and local density-functional theories. *J Chem Phys* 1993; **98**: 1372-1377.
5. Perdew JP, Wang Y. Accurate and simple analytic representation of the electron-gas correlation energy. *Phys Rev B* 1992; **45**: 13244-13249.
6. Schäfer A, Huber C, Ahlrichs R. Fully optimized contracted Gaussian basis sets of triple zeta valence quality for atoms Li to Kr. *J Chem Phys* 1994; **100**: 5829-5835.
7. Grimme S, Antony J, Ehrlich S *et al.* A consistent and accurateab initioparametrization of density functional dispersion correction (DFT-D) for the 94 elements H-Pu. *J Chem Phys* 2010; **132**: 154104.
8. Grimme S, Ehrlich S, Goerigk L. Effect of the damping function in dispersion corrected density functional theory. *J Comput Chem* 2011; **32**: 1456-1465.
9. Marenich AV, Cramer CJ, Truhlar DG. Universal Solvation Model Based on Solute Electron Density and on a Continuum Model of the Solvent Defined by the Bulk Dielectric Constant and Atomic Surface Tensions. *J Phys Chem B* 2009; **113**: 6378-6396.
10. Kresse G, Hafner J. Ab initio molecular dynamics for liquid metals. *Phys Rev B* 1993; **47**: 558-561.
11. Kresse G, Furthmüller J. Efficiency of ab-initio total energy calculations for metals and semiconductors using a plane-wave basis set. *Comput Mater Sci* 1996; **6**: 15-50.
12. Kresse G, Joubert D. From ultrasoft pseudopotentials to the projector augmented-wave method. *Phys Rev B* 1999; **59**: 1758-1775.
13. Eiden P, Liu Q, Zein El Abedin S *et al.* An Experimental and Theoretical Study of the Aluminium Species Present in Mixtures of $AlCl_3$ with the Ionic Liquids [BMP]Tf_2N and [EMIm]Tf_2N. *Chem Eur J* 2009; **15**: 3426-3434.
