# Supplementary material for: Unveiling cathode catalysis of fluorinated electrolyte additives for high-performance Na-Cl2 batteries
Source: Natl Sci Rev. 2025 Aug 12;12(10):nwaf333. doi: 10.1093/nsr/nwaf333 (PMC12491998; doi:10.1093/nsr/nwaf333)
Supplement: nwaf333_Supplemental_Files [file nwaf333_supplemental_files.zip › Teaser text.docx]

Fluorine-containing additives in sodium–chlorine batteries can form an efficient cathode catalyst to improve rate and cycling performance, opening a new avenue for high-rate, long-life rechargeable batteries.
